# Unfolding mechanism of thrombin-binding aptamer revealed by molecular dynamics simulation and Markov State Model

**DOI:** 10.1038/srep24065

**Published:** 2016-04-05

**Authors:** Xiaojun Zeng, Liyun Zhang, Xiuchan Xiao, Yuanyuan Jiang, Yanzhi Guo, Xinyan Yu, Xuemei Pu, Menglong Li

**Affiliations:** 1 Faculty of Chemistry, Sichuan University, Chengdu 610064, People’s Republic of China

## Abstract

Thrombin-binding aptamer (TBA) with the sequence 5′GGTTGGTGTGGTTGG3′ could fold into G-quadruplex, which correlates with functionally important genomic regionsis. However, unfolding mechanism involved in the structural stability of G-quadruplex has not been satisfactorily elucidated on experiments so far. Herein, we studied the unfolding pathway of TBA by a combination of molecular dynamics simulation (MD) and Markov State Model (MSM). Our results revealed that the unfolding of TBA is not a simple two-state process but proceeds along multiple pathways with multistate intermediates. One high flux confirms some observations from NMR experiment. Another high flux exhibits a different and simpler unfolding pathway with less intermediates. Two important intermediate states were identified. One is similar to the G-triplex reported in the folding of G-quadruplex, but lack of H-bonding between guanines in the upper plane. More importantly, another intermediate state acting as a connector to link the folding region and the unfolding one, was the first time identified, which exhibits higher population and stability than the G-triplex-like intermediate. These results will provide valuable information for extending our understanding the folding landscape of G-quadruplex formation.

G-quadruplex^1^,^2^ is guanine-rich nucleic acid sequence that can fold into a four –stranded structure. Four guanine bases can self-assemble through Hoogsteen hydrogen bonding to form a square planar structure called a guanine tetrad, and two or more guanine tetrads can stack on top of each other to form a G-quadruplex. The G-quadruplex structure can be further stabilized by presences of cations in the central channel between each pair of tetrads, such as K^+^, Na^+^ or Sr^2+^. The G-quadruplexes were found in biologically significant regions of the genome such as telomeres^3^,^4^,^5^ and immunoglobulin switch regions^6^,^7^. They can be formed of DNA, RNA, LNA, and PNA^8^,^9^,^10^ inside/outside the living cells and usually related with human diseases^5^,^11^,^12^. Therefore, structural stability and other aspects of physical chemistry of the G-quadruplexes have been important topics for the pharmacological industry and modern biology.

Most known G-quadruplex structures were obtained by NMR techniques. However, although NMR techniques greatly enhanced our understanding of the structures, the underlying molecular mechanism of the dynamical variation remains unclear, for example, the folding and unfolding kinetics, which play an important role in the biological function of the G-quadruplex. Several other biophysical techniques (mainly spectroscopies) were utilized to study the thermodynamic and folding stabilities of the G-quadruplexes, for example, circular dichroism (CD)^13^,^14^,^15^, UV absorption spectroscopy^13^,^16^,^17^ and NMR^18^. However, the folding/unfolding mechanisms have not been satisfactorily elucidated due to the ultrafast dynamics and microscopic nature of the issue. Many unanswered questions still remain as to how quadruplex structures unfold, how many metastable intermediates exist in the unfolding pathway and what their structures are. So far, there have been no consensus conclusions. The two-state unfolding model^19^ and multi-state one were proposed^20^. Consequently, it is highly desired to further study and cast light on the unfolding mechanism of the G-quadruplex.

Thrombin-binding aptamer (TBA) with a sequence of 5′GGTTGGT-GTGGTTGG3′ is the simplest G-quadruplex and is involved in the cascade of blood coagulation, thus, attracting considerable interests from experiments and theories. Some works from NMR^21^,^22^,^23^,^24^, X-ray crystallography^25^,^26^,^27^ already well characterized TBA structure, revealing that it can fold into an anti-antiparallel chair-like quadruplex structure which contain two guanine tetrads with a TGT loop and two TT loops, as shown in Fig. 1. Mao^18^ used NMR technique to study the stability of H-bonding of TBA and proposed an assumption concerning the unfolding process based on the hydrogen exchange rate that the TBA first uncouples the three base pairs: G1–G15, G2–G14 and G5–G11 and then opens the TGT loop, finally opens the TT loops and the sequence becomes an unstructured random coil. However, the macroscopic NMR technique could not provide atomic detail concerning the unfolding process and important intermediates for TBA. Molecular dynamics (MD) simulations have been wildly used to complement experiment to gain insight into the structure stability and the dynamics behavior in the folding/unfolding process^28^,^29^,^30^,^31^. Using classical molecular dynamic simulation, Wennmohs^32^ studied the structure–activity relationship of TBA and its caged variant. Reshetnikov^33^ utilized MD simulation to study TBA-thrombin complex and discuss the stabilization factors. Recently, Antonio^34^ combined NMR, CD and metadynamics methods to study the folding of TBA and confirmed the G-Triplex intermediate existing in the folding process, which were already hypothesized in other investigations on G-quadruplexes^35^. As reported by the experiments, the unfolding processes of G-quadruplexes generally occur at millisecond or second scale^36^,^37^, which are hardly accessible for equilibrium MD simulations. Thereby, some classical MD simulations were generally utilized to study TBA structure while the folding and unfolding processes were concerned with aid of some unequilibrium MD techniques. Eunae Kim^19^ utilized replica exchange molecular dynamics (REMD) simulation in combination with free-energy landscapes to investigate the folding process of TBA and proposed that the process follows a two-state folding behavior with a barrier of 6 kcal/mol. Pak^38^ used MD simulation in conjunction with PMF calculation to study the unfolding of TBA with inclusion of Sr^2+^. Their results indicated that the unfolding process exhibited a multiple stepwise behavior associated with the interplay of the metal ion and the uptake of water molecules. Recently, Zhang^39^ used steered molecular dynamics simulations to investigate the unfolding process of native TBA and obtained some similar observation to those of NMR experiments^18^. However, as known, although the unequilibrium simulations could cross high energy barriers between the native state and the unfolding one and enhance sampling of conformations, they would cause some deviations from the real unfolding pathway. In addition, although the previous MD studies also provided structure information regarding the conformation variation in the unfolding pathway, these conformations were almost simply selected from MD trajectories and were not ensured to be important meta-intermediates. Thus, some important problems regarding the number of intermediates, their structures and the transitions between them still remains an unattained goal.

Markov state model (MSM) is a kinetic network model that could partition conformation space into discrete states and obtain their kinetics through calculating transition probabilities between the states^40^,^41^. Consequently, MSM could predict long timescale events from multiple short MD simulations. This method has been promising tool to explore the complete energy landscape of biomolecules. A number of successful examples of applying MSM have already been reported in the field of protein and DNA/RNA folding^42^,^43^,^44^,^45^. With aid of MSM, Pande performed molecular simulation of *ab Initio* protein folding for a millisecond folder NTL9(1–39), giving its folding pathway^46^. Noé utilized MSM to study the folding mechanism of PinWW protein^47^. Yao combined MD and MSM method to study ligand induced change of β_2_AR from active conformation to inactive one^48^. Huang constructed Markov state models from extensive all-atom molecular dynamics (MD) to study dynamics of pyrophosphate ion release^49^,^50^ and translocation^51^ for RNA Polymerase II, the metastable conformational states of the hIAPP monomer and the dynamics of transitioning between them^52^.

Hence, in order to address the problems aforementioned, we, herein, combined MD and MSM to in depth study the TBA unfolding mechanism. First, we performed high-temperature MD to obtain initial seeds in the unfolding conformation space. Based on thirty-one representative seeds, 100-ns conventional MD was further carried out for every seed at ambient temperature. Then, MSM was utilized to gather all short trajectories together and identify metastable intermediates and transition pathways between them. Our results show that there are multiple unfolding pathways with multi-state intermediates for TBA, in which one G-triplex-like intermediate was also observed. More importantly, another important intermediate, through which most unfolding pathways pass was first time identified to act as a connector role to link the folding region and the unfolding one in the unfolding process. These observations provide valuable information for extending our understanding of the unfolding mechanism of the G-quadruplexes.

## Results and Discussions

### MD simulation in high temperature

**Figure 2:**
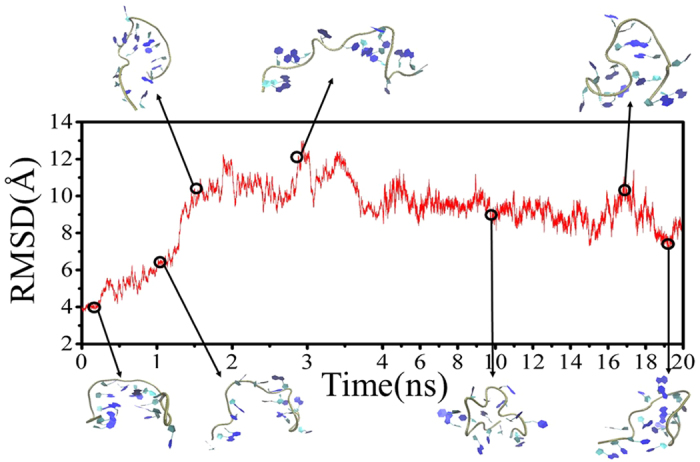
The changes in the RMSD values of backbone atoms of TBA with respect to the crystal structure and structures of some representative snapshots.

In order to obtain initial seeds in the unfolding conformation space, three parallel 20-ns simulations with different starting velocities were first performed for TBA. Similar results were observed. Figure 2 representatively shows relative mean standard deviation (RMSD) values of TBA backbone atoms within 20 ns simulation time at 498 K temperature for one trajectory, with respect to the TBA crystal structure. As can be seen from Fig. 2, the RMSDs quickly increase up to 13 Å within first 2.5 ns and then fluctuate around 8−13.5 Å at the remaining simulation time. Figure 2 also shows some representative conformations in the trajectory. These conformations clearly show that TBA almost achieve the unfolding state close to 3 ns at the high temperature of 498 K, after which the unfolding-state also occurs to some extent rearrangement. In terms of RMSD values, we clustered the conformations from the three parallel trajectories into 31 different groups and selected one representative conformation from every group as initial seeds (vide 31 structures) to further perform 100-ns equilibrium MD simulations under ambient temperature. The representative conformation has the lowest RMSD value with respect to the average structure of each cluster.

### Markov State Model (MSM) analysis

**Figure 3:**
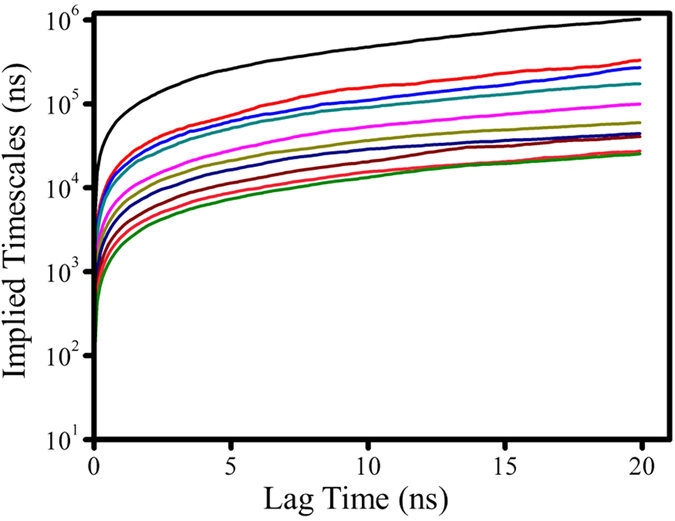
Top ten implied timescales as a function of the lag time, derived from the transition probability matrix with all 231 states.

**Figure 4:**
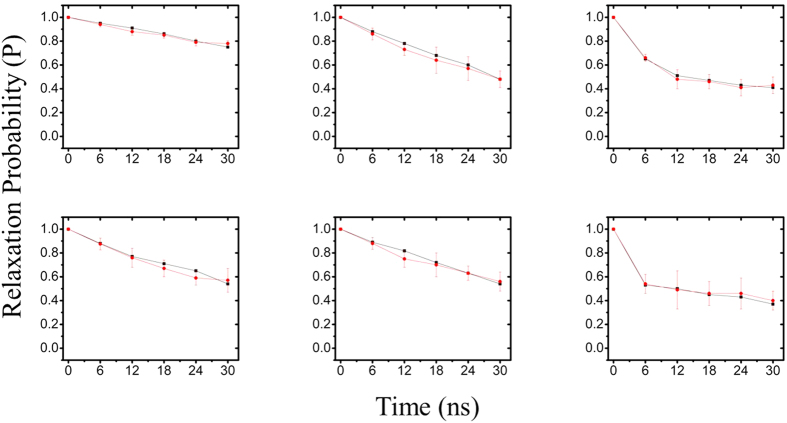
‘Residence probability tests’ for six highly-populated macrostates, which show the probability to stay at a certain macrostate as a function of the propagation time. The red curves come directly from the MD simulations while the black curves are predicted from the MSM with a lag time of 6 ns.

**Figure 5:**
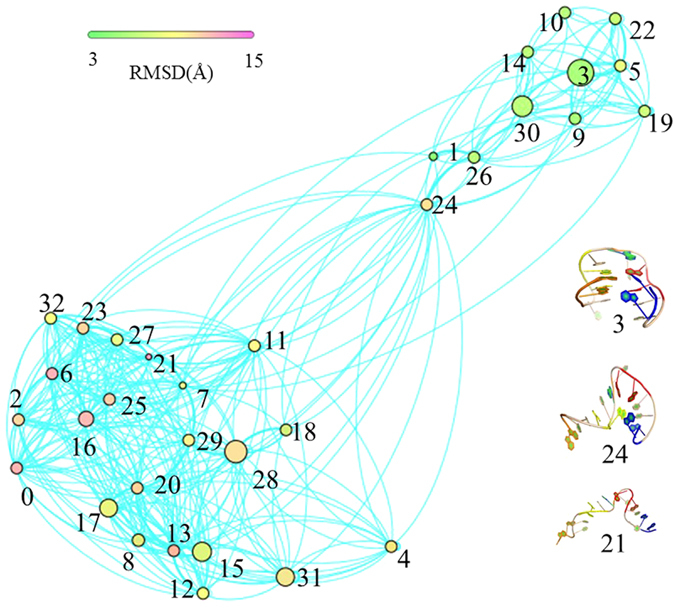
Unfolding network based on 32 macrostates. The sizes of nodes are proportional to the corresponding equilibrium populations and nodes are colored according to the average backbone RMSD from the crystal structure. Representative structures of state 3, state 6 and state 21 are shown in right, which are colored by the different guanines (blue for G1 and G2, red for G5 and G6, yellow for G10 and G11, orange for G14 and G15 and green for G8).

MSM was first built from 31 100-ns MD trajectories by means of K-center clustering algorithm, through which all MD conformations were divided into 231 microstates. As known, if a system is Markovian, the implied timescales will not change with lag time. Otherwise, the implied timescales of non Markovian would increase quickly with increasing the relaxation time. Thereby, we checked changes of the implied timescales of our models, as shown in Fig. 3. It can be seen that the relaxation timescales almost approach to equilibrium at about 6 ns, confirming that our system is Markovian. Then we selected the lag time of 6 ns as the time ***τ*** to construct our Markov States Model, in which robust Perton Cluster Cluster Analysis (PCCA+) was applied to lump the 231 microstates into 32 macrostates. In order to further validate the reliability of the MSM constructed, we also performed residence probability test^47^,^53^ for six highly-populated macrostates (vide Fig. 4). It is clear that the probability predicted from the MSMs for a given macrostate to stay with it after 6-ns lag-time can well reproduce the direct counts from the MD simulations, further confirming that our MSMs are consistent with the original dataset from which they were constructed. Then, we construct a transition network between the 32 macrostates using Gephi software^54^, as shown in Fig. 5. The size of the nodes in Fig. 5 reflects the population of conformations in each state and edges reflect that two states connected have conformation transition in the relaxation time. As can be seen from Fig. 5, the node of 3 contains native-like conformations with the smallest RMSDs while the conformations in the node of 21 with the largest RMSDs contain unfolding-like structures, thus representing the unfolding-like state. In addition, it is clear that the network exhibits two main regions: the dense region around the state 21 at the bottom and the sparse one around state 3 at the top. Our calculation results show that the RMSDs of back-bone atoms in the sparse part are lower than 8 Å and the distances between 3′ end and 5′ one is lower than 15 Å while the two values in the dense network part are in the ranges of 8–13 Å and 15–43 Å, respectively. Thereby, the sparse part of network should be assigned to the folding-like states and the dense one should belongs to the unfolding-like states. The two parts are well-separated and connected by several intermediate nodes. A further inspection shows that most pathways pass through the node of 24, indicating that the node is an obligatory and gatekeeper state. In addition, it is observed that there are less nodes in the sparse region than the dense one. Based on the topology, it can be assumed that more frequent transitions occur in the unfolding-like region while the folding-like region has less transitions and exhibit higher stability. In a whole, the network reveals that there are multiple routes in the unfolding process.

**Figure 6:**
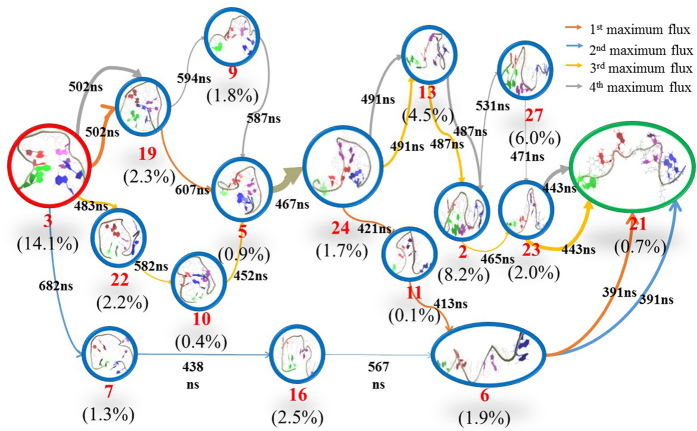
Unfolding pathways for four maximum fluxes from state 3 to state 21. Arrow colors denote different pathways and the weight of arrow indicates the transition probability between states. The representative structure in each state is colored by the different guanines (blue for G1 and G2, purple for G5 and G6, red for G10 and G11 and green for G14 and G15). The population of each state is listed in parenthesis. Transition times between two states are derived from MFPT calculation.

**Figure 7:**
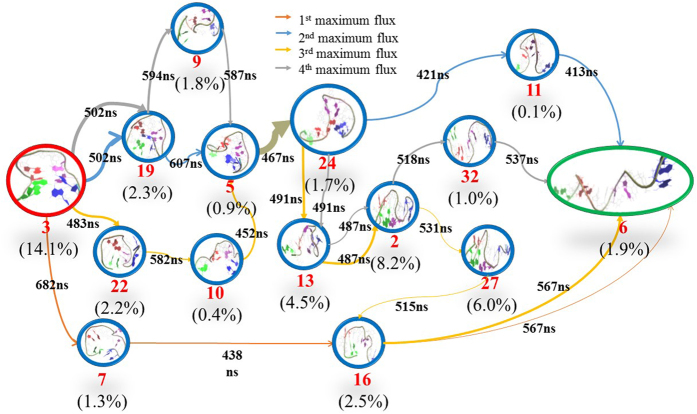
Unfolding pathways for four maximum fluxes from state 3 to state 6. The Other details are same as Fig. 6.

In order to gain insight into the unfolding mechanism, we used Transition Path Theory (TPT)^47^,^55^,^56^ method to obtain the unfolding pathways. As accepted, the most prominent difference between the native state and the unfolded one is the existence of G-quadruplex or not. Thereby, we selected state 3 as a native-like folding state due to the existence of G-quadruplex and its smallest RMSD value to the native structure. While for many states of 31 macrostates, the native G-quadruplex structures were already broken. Since TBA is a single-strand DNA with a sequence of 5′GGTTGGT-GTGGTTGG3′, the fully unfolding state should extends to one linear-like structure with full-separation of 5′-end and 3′-end. Thus, we selected state 6 and state 21 as the unfolding states (viz., terminal states) since their structures are close to the linear-like ones and have the highest RMSD values with more than 12 Å and the largest distances between the two end residues (viz., G1 and G5) and between the eight guanines consisted of the G-quadruplex structure. The differences in RMSD values between state 6 and state 21 is very small, only 0.3 Å. The distances between the two end residues are more than 43 Å (44.3 Å in the state 6 and 43.3 Å in the state 21). Table 1 and Figs 6 and 7 show four highest fluxes in two cases (viz., the unfolding from state 3 to state 21 and one from state 3 to state 6). In addition, we also calculated Mean First Passage Time (MFPT) for these pathways, as depicted in Figs 6 and 7. A careful inspection on Table 1 indicates that the first and the second highest fluxes in the unfolding pathway from state 3 to state 6 are similar to the second and the first highest fluxes from state 3 to state 21, respectively. Thereby, we mainly focused on the two fluxes to discuss the unfolding mechanism of TBA in the work. The unfolding in the first pathway starts from state 3, then goes through state 19, state 5, state 24, state 11, state 6, and finally ends in the unfolding state 21. As revealed from Fig. 5, states 3, 19, 5 are located in the folding-like region while the states 11 and 6 are located in unfolding-like one. The state 24 acts as a connector. Compared with the first pathway, the second unfolding pathway exhibits a simpler process with less intermediates, in which TBA unfolds from state 3, then gradually passes through state 7, state 16, state 6 and finally achieves the state 21. The results of MFPT reveal that the first pathway presents about 2.81 μs unfolding time, slightly longer than the 2.07 μs of the second pathway, despite of less intermediates in the second pathway. The unfolding times of the two pathways almost fall within the time scale of experimental estimations on some G-quadruplexes with the unfolding rates of 10^−5^ s^36^,^37^.

As revealed, the TGT loop can protects the upper G-quadruplex and controls ions in or out, while the TT loop is mainly responsible for distinguishing and binding thrombin. Therefore, in the following discussions, we mainly focus on the changes in hydrogen bonding and distances between the eight guanines forming the G-quadruplex, in particular for G1 at 5′ end and G15 at 3′ end. Tables 2 lists the distances between the residues mentioned above and the centroid of TBA, the distances between these residues, RMSDs of TGT and TT loops, which were obtained through averaging the results from 100 representative structures for each state.

### First unfolding pathway from state 3 to state 21

**Figure 8:**
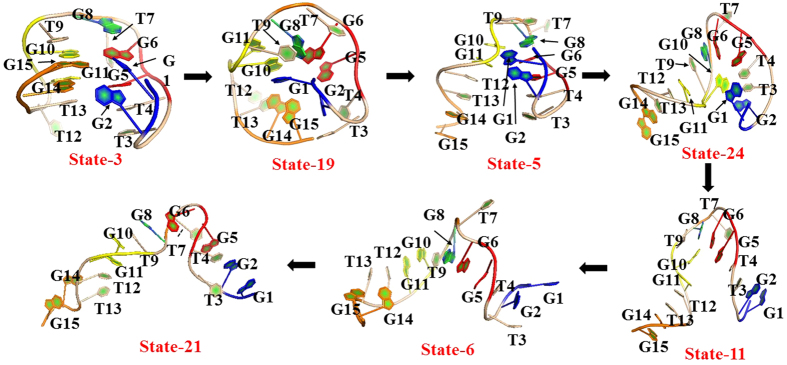
The representative TBA structures of seven macrostates in the unfolding pathway from state 3 to state 21 for the maximum flux. The structures are colored by the different guanines (blue for G1 and G2, red for G5 and G6, yellow for G10 and G11, orange for G14 and G15 and green for G8).

The representative conformation of each state in the first unfolding pathway is shown in Fig. 8.

**State 3**: Although the distances between the two end-residues (viz., G1 and G15) and the centroid of TBA in the state nearly have no changes with respect to the native structure, the conformation in Fig. 8 shows that they twist inward, leading to a significant drop in the distance of G1–G15 from 11.7 Å of the crystal structure to 8.2 Å and the disruption of H-bonding of G1–G15, as shown in Table 2. However, the H-bonding between G6 and G10 residues still remains. In addition, T9 residue in the TGT loop exhibits a leave from the centroid with the distance change from 8.4 Å to 10.7 Å while the distances between G8 and T7 residues in TGT loop and the centroid of TBA (labelled as C) nearly unchanged. Two new H-bonds are formed between TGT and the upper G-quadruplex plane (viz., T9…G10, G8…G10). For the four guanines in the lower quadruplex plane, no obvious change is observed in the distances between them and the centroid. As a result, H-bonds between the four guanines are all reserved in the state 3, as shown in Table 3. For the TT loops, the distances between the TT residues and the centroid increase from 11.0 Å to 14.8 Å for T3-C Å and from 8.2 Å to 9.2 Å for T4-C, resulting from the twisting of G1 residue. The observations indicate that the TT loop in the left hand leaves from the centroid. Whereas for the TT loop in the right hand, the relative small changes are observed. The conformation variation in the TT loop induces the formation of the four new H-bonds involved in T4…G5 and T12…G11. In a whole, the state 3 still has many H-bonds through reserving some original H-bonds in native state and forming some new H-bonds. Thus, the native-like macrostate should be relatively stable.

**State 19**: In the state, the distance between G1 residue and the centroid of TBA decreases from 7.1 Å to 3.9 Å (see Table 2), indicating that the G1 further twists inward. As further evidenced by Fig. 8, G1 residue already inserts into the center of TBA, close to G10 residue. The insertion of G1 significantly loosens the conformation around 3′ end of TBA, further breaking the hydrogen bond between G11 and G14 existed in the state 3 (see Fig. 8). Accordingly, G15 starts to move outside since the distance of G15-C increases from 7.5 Å to 10.0 Å. Consequently, the distance of G1–G15 increases from 8.2 Å in the state 3 to 10.5 Å of the state. In addition, the inward movement of G1 causes the residues G2, G3, G4 and G5 to twist outward (see Table 2 and Fig. 8), resulting in the disruption of H-bonds between the guanines in the lower plane and the increase in the distance between the two TT loops. In terms of the distances between the TGT loop and the centroid of TBA and the RMSD value of TGT (see Table 2), TGT loops does not exhibit significant variation. The loosing of 3′ end structure causes T12 and T13 residues moving away from the centroid, as evidenced by the increases in the distances of T12-C (from 10.7 Å to 12.6 Å) and T13-C (9.0 Å to 11.7 Å) with respect to the previous state. Consequently, six new hydrogen bonds are formed, including two H-bonds between T9 and G10, three H-bonds between G6 and G2 and one H-bond between G2 and G8. However, the hydrogen bonding between the guanine residues of the G-quadruplex plane are completely broken and the conformation of G-quadruplex is destroyed, as reflected by Table 2 and Fig. 8.

**State 5**: In the state, the distance of G15-C increases from 10.0 Å to 18.4 Å, indicating that the 3′-end is far away from TBA centroid, which also brings the G14 residue away from the centroid, as evidenced by the increase in the distance of G14-C from 10.6 Å to 15.5 Å. Accordingly, the distance of G2–G14 is increased from 12.4 Å to 21.5 Å. The change also loosens the local structure around G10 residue. On the other hand, G1 also moves away from the centroid, as reflected by the increase in the distance of G1-C from 3.9 Å to 9.1 Å, which brings the G2 residue away. Thus, the distances of G1-C and G2-C are slightly larger than the value in the crystal structure. Vittorio Limongelli^34^ proposed a stable G-Triplex structure existed in the TBA folding process, in which stable H-bonding exist between the G1, G6 and G10 residues in the upper G-quarter and between G2, G5, G11 in the lower G-quarter. For the state 3 in the highest flux pathway, two H-bonds are formed between G2, G5 and G11 in the lower G-quadruplex plane (see Table 3). But, different from the G-Triplex structure, H-bonds are not observed between the G1, G6 and G10 in the upper G-quadruplex plane for the state 5. However, the distances of G1–G6 (9.7 Å) and G6–G10 (10.4 Å) are close to ones of G2–G5 and G5–G11 in the lower quadruplex plane, respectively. Furthermore, as can be seen from Fig. 8, the local structure of G1, G6 and G10 residues is similar to one of the G2, G5 and G11 residues in the lower quarter. Thus, it can be suggested that the state 5 should be G-triplex-like intermediate. The result is not unexpected. Fiasconaro used unequilibrium MD simulations with pulling force to study the mechanical unfolding of some DNA and RNA G-quadruplexes and found that the triplex state would has different arrangements depending on the presence or not of the ions^57^. Indeed, in the work, the elimination of H-bonds between G1, G6 and G10 residues of TBA to large extent stems from that the removal of the central ions reduces the geometrical constraints in the plane formed by the three residues. Compared to the state 19, the TGT loop and T3T4 loop nearly remain unchanged in the state, judged from their RMSD values. However, the structure of T12T13 loop occurs significant changes. The distance of T12-C decreases from 12.6 Å to 9.6 Å. In addition, Fig. 8 clearly shows that the backbone of T12T13 loop to some extent moves outside with respect to the previous state. In brief, the state mainly exhibits the unfolding of 3′-end and significant separation between G1 and G15.

**State 24**: In the state, the distances of G1-C and G15-C change very slight compared with the previous state. The distance of G1–G15 only changes from 24.0 Å to 26.5 Å. The observation indicates that TBA doesn’t further display significant unfolding. However, significant variation in the local structure still be observed. The distance of G10-C decreases from 6.2 Å to 2.9 Å. As further revealed by Fig. 8, G10 already enter into between G2 and G6 residues, leading to increases in the distances of G2-C, T3-C, T4-C, G5-C and G6-C with respect to the previous state, as can be seen from Table 2. Hydrogen bonds formed between G2, G5 and G11 in the previous state are all broken in the state. In the other words, the G-triplex-like structure were destroyed in the state. Alternatively, G10 surrounded by T3, T4, G5, G6 and G5 residues forms H-bonds with these residues. The inward extending of G10 also leads to tilt of TGT loop and T12T13 so that some changes occur for the distances of T9-C, G8-C, T7-C and T12-C, especially the distance of T9-C (from 8.1 Å to 3.1 Å). The change forces T9 and G11 closer, leading to the formation of two hydrogen bonds between the two residues. As shown in Table 3, more H-bonds are formed in the state with respect to the G-triplex-like state 5, which should significantly contribute to the stability of the state 24. Indeed, the state 24 has higher population than the state 5 (vide Fig. 6). As revealed by the transition network above (viz., Fig. 5), the state 24 acts as a connector in the transition pathway. Taken together, it is reasonable to assume that the state 24 should be a very important intermediate in the unfolding pathway, which was not identified and reported by previous studies. One possible reason may be due to that the absence of centroid ion would induce more structural rearrangement, thus leading to existence of some important metastable intermediates, which are most possibly relevant with the late stage of unfolding, as proposed by Šponer who studied the unfolding of G-quadruplex without no salt^58^. In brief, the structure variation in the state 24 mainly stems from inward extending of G10 residue, which breaks the structure around 5′-end, leading to significant changes in the structure of TBA with RMSD up to 10.3 Å.

**State 11**: In the state, the G1 residue in the 5′ end also starts to extend outward since the distance of G1-C increases from 9.8 Å to 13.9 Å while the distance of G15-C almost remains unchanged. In addition, the distance of G1–G15 still keeps at ~26 Å while the distance of G2–G14 decreases from 26.9 Å to 25.7 Å, indicating that 3′-end and 5′ end in the state do not further separate. However, due to the outward extending of G1, the local structure involved in T3, T4, G5 and G6 residues become loosen, leading to disappearance of the H-bonding between G10 and these residues existed in the previous state. Accordingly, the G10 residue leaves from the centroid of TBA so that the distance of G10-C increases from 2.9 Å to 7.2 Å. The data in Table 2 associated with the distances between the residues in the two TT loops indicate that the two TT loop are closer to the centroid of TBA with respect to the previous state. As can be seen from Fig. 8, the residues around TGT loop are still relatively compact. Compared to the state 4, the H-bonding between G10, G6 and G11 still exist and some new H-bonds are formed for G10…G8 and G6…T7. However, the number of H-bonds in the state are significantly decreased with respect to the previous sate (see Table 3), implying a weakness in its stability, thus leading to its very low population (0.1%) and relatively short MFPT (413 ns) from it to the next state 6. In a whole, the TBA in the state displays a U-like conformation (see Fig. 8), in which the 3′-end starts to significantly extend outward in this state.

**State 6**: In the state, the 5′-end and 3′-end of TBA simultaneously and remarkably extend outward. The distance of G1-C increases from 13.9 Å to 22.3 Å, and the distance of G15-C rises from 18.4 Å to 22.8 Å, leading to a significant rise in the distance of G1–G15 from 26.9 Å to 44.4 Å and displaying line-like structure. Although the H-bonds near the TGT loop existed in the previous state are all broken, T7 residue forms H-bonding with G6 and G5 residues. Consequently, the local structure around T7 is still relatively compact. But, the overall structure already extends into line-like conformation. The total RMSD increases from 8.7 Å in the previous state to 12.6 Å in the state.

**State 21**: Similar to the state 6, the state 21 is also a linear-like structure. Its RMSD is only slightly lager by 0.3 Å than the state 6. However, there are still some differences in local structure between the two states. For example, T7 residue twists inward so that the distance of T7-C decreases from 9.4 Å to 8.2 Å, leading to disruption of H-bonding between T7, G5 and G6, which exists in the state 6. As a result, the relatively compact structure around T7 is damaged in the state, as can be seen from Fig. 7. However, there are two new H-bonds formed between T13, T12 and G14 so that the structure around T13 becomes relatively compact. In a whole, the states 6 and 21 almost unfold into linear-like structures, in which the linear backbones would randomly form a few hydrogen bonds so that the unfolded states hardly keep a completely linear conformation. In addition, MFPT results reveal that there is the second shortest transition time (391 ns) for the change from state 6 to state 21, implying their relatively quick transition.

### The Second Pathway from state 3 to state 21

**Figure 9:**
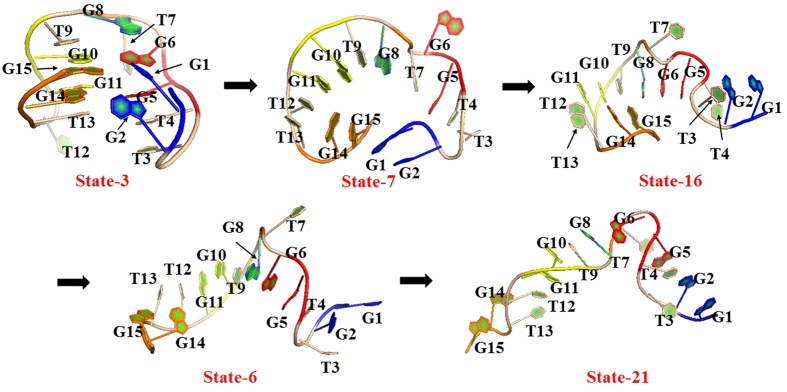
The representative TBA structures of five macrostates in the unfolding pathway from state 3 to state 21 for the second maximum flux. The other details are same as Fig. 8.

As observed above, the second unfolding pathway exhibits a simpler process with less intermediates than the first pathway. Furthermore, the pathway does not pass through the G-triplex-like state (state 5) and gate-keeper one (state 24) but directly transits from the state 7 in the folding-like region to the state 16 in the unfolding-like region. Figure 9 shows the representative conformations in the pathway. The first state (viz., state 3) in the second pathway is same as the first pathway.

**State 7**: In the state, the 3′-end residue (viz., G1) and the 5′-end one (viz., G15) leave away from the centroid, as evidenced by data in Table 4, leading to their separation. Their distances are close to those of the states 19 and 5 in the first pathway. However, as can be seen from Table 4, the distances of T3–T13, T4–T12, G5–G11, and G6–G10 significantly increase and the distances between T4, G5, G6, G11, T12, T13 residues and the centroid of TBA all increase by more than 2 Å with the maximum of 6.8 Å. These distances above are larger than the state 5 and close to the state 21 in the first pathway. Due to the opening of the two TT loops, the H-bonds in the G-quadruplex are all broken. However, alternatively, two new H-bonds form between G2 and G5, and between G14 and G11. G14 also forms four hydrogen bonds with T12 and G10 residues, as reflected by Table 5. The new hydrogen bonds formed between T7 and G8 cause the RMSD of TGT loop to increase to be 4.4 Å. These H-bonds should contribute to the stability of the intermediate. As shown in Fig. 9, for the state 7, the structure of the backbone also displays a ring-like conformation, in which G1 and G15 are located in the gate of this ring. The backbone RMSD increases from 5.0 Å to 8.5 Å, close to the state 5 in the first pathway.

**State 16**: Compared to the previous state 7 located in the folding-like region, the state is located in the unfolding-like one, indicating that it is a direct transition from the folding-like region to the unfolding-like one without passing one gate-keeper intermediate like state 24. Surprisingly, the transition time derived from MFPT is only 436 ns between state 7 and state 16, shorter than those between the folding-like states in the first pathway, in turn implying the likelihood of the unfolding transition.

As can be seen from Table 4, compared with the state 7, the distances of G1-C and G15-C remarkably increases from 10.2 Å to 17.5 Å and from 8.8 Å to 12.5 Å, respectively, leading to the significant separation between G1 and G15 from 11.1 Å to 27.2 Å. These values are close to those of the fifth state (viz., state 11) in the first pathway. Due to the separation of the two ends, the distance of G2–G14 also increases up to be 26.1 Å, larger than that of the state 11 in the first pathway. Consequently, the H-bonds existed in the previous state are all broken. However, the G6 residue forms three hydrogen bonds with G8 and two H-bonds with G2 (vide Table 5). At the same time, G2 and T7 forms one hydrogen bond with T4 and G5, respectively. For the TGT and TT loops, no obvious changes are observed with exception of outward twist of T7 residues. In a whole, the state is similar to the state 11 in the first pathway but exhibits more H-bonds, implying its relative stability with 2.5% population.

The fourth state (viz., state 6) and the last state (viz., state 21) are the same as the first pathway. G1 and G15 further extend outside, and TGT and two TT loops also completely open. Finally, TBA finishes unfolding.

## Conclusions

The unfolding mechanism of TBA could provide important information for understanding the structure and the function of DNA and RNA G-quadruplexes. Although some unequilibrium MD works already focused on the issue and provided valuable information, the restrained methods to some extent limit insight into the real conformation space in the unfolding pathway. Thus, in the work, we combined MSM and extensive MD simulations to in depth study the unfolding mechanism of TBA. Some novel and different observations were obtained from the work.

Our results revealed that there are multiple pathways in the unfolding of TBA. Some pre-folding and pre-unfolding intermediate states were identified and characterized. It was noteworthy that most pathways would pass through two obligatory intermediate states. One state is located in the pre-folding region and its structure is similar to the triplex-structure existed in the cation-driven folding process for the G-quadruplexs and TBA, but the triplex-like intermediate is lack of H-bonding between guanines in the upper plane. More importantly, it is the first time to identify another important state (viz., state 24), which acts as a gate-keeper or connector in the pathway from the folding region to the unfolding one. The connector state was not reported previously, which exhibits relatively stable structure stemmed from relatively many H-bonds in the state.

The highest flux pathway further supported the main process of the TBA unfolding proposed by NMR experiment, viz., the unfolding started from disruption of the H-bonding of G1–G15, G2–G14, then uncoupling interactions of G5–G11 and G6–G10, finally unfolding. However, our result indicated that the 3′ end is prior leave from the center of TBA to the 5′ end, different from the observation from Zhang^39^ (SMD) and providing a further support for the observation from Vittorio^34^ and Pak^38^ works.

In addition, one simpler pathway was revealed, involving less intermediate states and exhibiting significant differences from most pathways. In the pathway, one pre-folding state directly transit to the pre-unfolding one without passing through G-triplex-like and gatekeeper intermediates. Furthermore, the direct transition seems not to exhibit higher barrier than the most transition existed in the pre-folding region, judged from the MFPT time calculated. These observations provide more valuable information for understanding the stability and the function of G-quadruplexes.

### Computational methods

#### Molecular dynamics simulation at high temperature

All the MD simulations were performed using AMBER version 12^59^ with the force field Amber99_parmbsc0^60^ for the calculation of bonded terms and non-bonded ones. In this study, we first applied MD simulation at high temperature to obtain the initial thermodynamic sampling. The initial structure of 15-TBA was taken from PDB entry 148D^61^, the twelfth frame. The system was neutralized with 14 Na^+^ and surrounded by 10 × 10 × 10 Å^3^ TIP3P^62^ water box with 4747 water molecules. Consequently, the system includes 14743 atoms in total. The system was equilibrated by energy minimization with steepest algorithm for 100ps in NVT ensemble first. Then the system was equilibrated for 200 ps in NPT ensemble and warmed to 498K within 200 ps. Three parallel 20-ns MD simulations were performed at 498K and 1 bar in NVT ensemble. The integration step for MD simulations was set to 1 fs. The particle mesh Ewald (PME) method^63^ was applied to treat long-range electrostatics interactions with a 12 Å non-bonded cutoff. All bond lengths involving hydrogen atoms were constrained by using the SHAKE algorithm^64^ with a tolerance of 1.0 × 10^−5 ^Å. The atomic coordinates were saved every 1 ps for further analysis.

#### Molecular dynamics simulation at ambient temperature

To select initial conformations from the high-temperature MD for further equilibrium MD simulations at ambient temperature, we first divided all the high-temperature MD conformations into 31 clusters using the k-centers clustering algorithm. We then chose the center conformations of each cluster and initiated 100-ns MD simulations at 298K with the similar setup as described above. Finally, we have collected 3.1 μs of simulation trajectories in total. All of the trajectories were analyzed using the analysis module of AMBER 12.0 and some other developed specific MD analysis programs.

### MSM building

Markov state model (MSM) is defined by a serial of states and transitions probability between these states. It can be constructed by the following three steps:

1. Assign all conformations into different states by clustering methods. This step can be summarize as **X**_***t***_ → ***s***(***t***). **X**_***t***_ represents all the conformations and ***s***(***t***) represents state which the conformation was aligned in at time ***t***.
2. Define a transformation between state ***i*** and ***j*** state as ***s***(***t***) = ***i***, ***s***(***t*** + ***τ***) = ***j*** at ***t*** and relaxation time ***τ***. Count all these transformations into a counting matrix ***C***, in which ***C***_***ij***_ represents the number of transformation between state ***i*** and ***j***. Here we use Maximum likelihood estimation (MLE) to calculate the counting matrix. Then, estimate transition probability matrix ***T*** according to ***C***, in which ***T***_***ij***_ represents the probability of transition between state ***i*** and ***j*** in relaxation time ***τ***.

Based on the strategy above, we first applied the k-centers clustering algorithm to divide all the MD conformations into 231 microstates based on the cutoff value of 0.75 Å. We lumped the microstates into 32 metastable macrostates using the Perron cluster cluster algorithm (PCCA+) and constructed 32-macrostate MSMs. We symmetrized and normalized the transition count matrix by column to obtain the transition probability matrix for this MSM. Using TPT, we calculated the fluxes for the different conformation transitions. The MSM construction was performed using the MSMBuilder2.0 software^65^,^66^,^67^.

### Transition Path Theory (TPT) analysis

We further used Transition Path Theory (TPT) to estimate the probability of these transition pathways (called as fluxes) in terms of the following method. If we define the start state as ***A***, the target state as ***B*** and the other states as ***I***, the forward committor probability 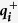 that the trajectory started in *I* state visits ***B*** before ***A*** can be estimated by Eq. (1):


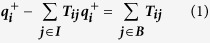


Accordingly, the probability of backward transition one can be given by Eq. (2):


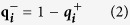


The connecting probability of different pathway can be obtained by calculating the fluxes between the different states. These fluxes can be defined as:


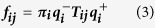


In Eq. (3), ***π***_***i***_ is the equilibrium probabilities. Actually, because ***f***_***ij***_ contains an incomplete loop, the entering probability 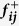 is calculated by Eq. (4):


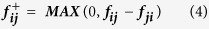


### Calculation of Mean First Passage Time (MFPT)

The Mean First Passage Time (MFPT) is defined as the average time for the transition between two states^65^. The MFPT from state ***i*** to state ***f*** can be obtained by solving the following set of equations:


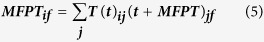


where ***T***(***t***)_***ij***_ is the transition probability from state ***i*** to state ***j***, ***t*** is the lag time that is equal to 6.0 ns in our model. For each transition, a set of linear equations can be solved under the boundary condition that ***MFPT***_***ff***_  = 0.

## Additional Information

**How to cite this article**: Zeng, X. *et al.* Unfolding mechanism of thrombin-binding aptamer revealed by molecular dynamics simulation and Markov State Model. *Sci. Rep.* **6**, 24065; doi: 10.1038/srep24065 (2016).

## Figures and Tables

**Figure 1:**
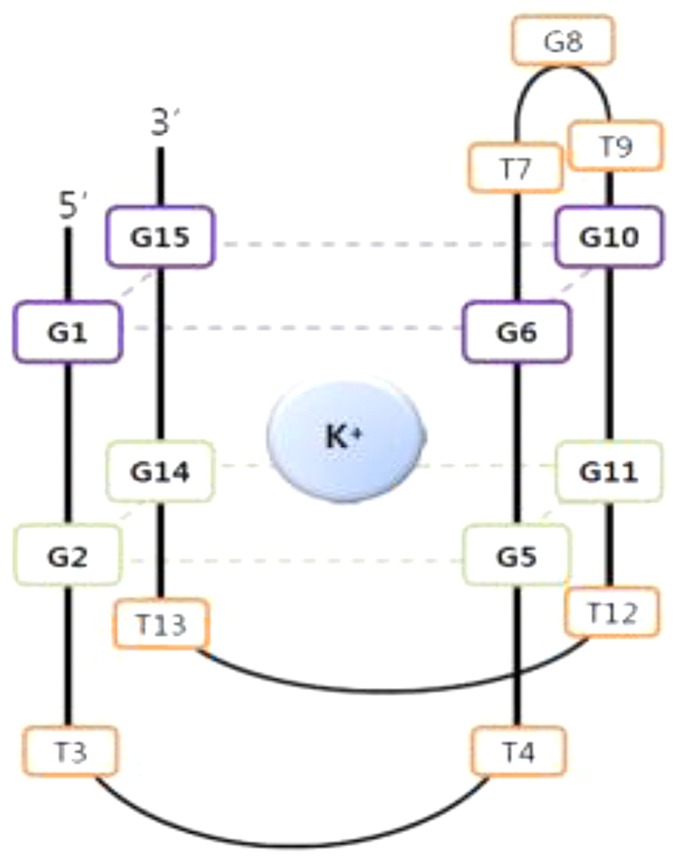
Schematic structure of thrombin binding aptamer TBA-15/K^+^ complex, quoted from ref. 19.

**Table 1:** Four maximum fluxes for two final states.

No.	State-3 as initial native-like structure and State-21 as final unfolding structure
1	3-19-5-24-11-6-21
2	3-7-16-6-21
3	3-22-10-5-24-13-2-23-21
4	3-19-9-5-24-13-2-27-23-21
	**State-3 as initial native-like structure and State-16 as final unfolding structure**
1	3-7-16-6
2	3-19-5-24-11-6
3	3-22-10-5-24-13-2-27-16-6
4	3-19-9-5-24-13-2-32-6

**Table 2:** Average RMSD values (in Å) with respect to the crystal structure and average distances (in Å) between residues^a^, derived from 100 structures of each state in the unfolding pathway from state 3 to state 21 with the maximum flux.

	148D^b^	State-3	State-19	State-5	State-24	State-11	State-6	State-21
RMSD/Å								
All^c^	0	5.0 ± 0.6	6.7 ± ± 0.6	8.5 ± 0.7	10.3 ± 0.7	8.7 ± 0.6	12.6 ± 0.6	12.9 ± 0.6
TGT d	0	3.7 ± 0.7	4.5 ± 0.7	4.4 ± 0.6	4.2 ± 0.6	3.9 ± 0.7	4.0 ± 0.6	3.8 ± 0.6
T3T4^e^	0	3.2 ± 0.6	3.3 ± 0.6	3.4 ± 0.7	3.4 ± 0.6	3.5 ± 0.7	3.4 ± 0.6	3.4 ± 0.6
T12T13^f^	0	3.7 ± 0.6	3.7 ± 0.6	3.7 ± 0.6	3.7 ± 0.6	3.7 ± 0.7	3.7 ± 0.6	3.5 ± 0.6
**Distance/**Å								
G1-C	7.6	7.1 ± 1.3	3.9 ± 2.0	9.1 ± 1.8	9.8 ± 2.0	13.9 ± 1.5	22.3 ± 2.0	21.7 ± 2.0
G2-C	8.2	8.3 ± 1.2	7.3 ± 2.1	9.2 ± 2.2	12.5 ± 2.4	13.6 ± 1.8	19.4 ± 2.2	18.0 ± 1.8
T3-C	11.0	14.8 ± 1.2	10.2 ± 1.6	10.9 ± 0.9	13.0 ± 1.3	11.8 ± 2.1	16.4 ± 1.6	17.4 ± 2.3
T4-C	8.2	9.2 ± 1.4	9.6 ± 1.2	10.1 ± 1.1	12.2 ± 1.1	10.2 ± 1.9	12.8 ± 1.6	13.1 ± 1.7
G5-C	7.2	7.5 ± 1.2	10.2 ± 1.6	8.0 ± 1.9	10.3 ± 1.3	9.0 ± 1.5	9.8 ± 0.9	10.9 ± 1.4
G6-C	7.3	7.3 ± 1.0	9.1 ± 1.4	7.42 ± 1.8	9.0 ± 0.9	9.5 ± 0.9	6.7 ± 1.3	8.4 ± 2.0
T7-C	10.7	10.7 ± 1.6	12.9 ± 1.8	14.4 ± 1.8	13.3 ± 1.7	132 ± 1.6	9.4 ± 1.8	8.2 ± 1.5
G8-C	8.1	8.0 ± 0.9	8.2 ± 0.9	8.5 ± 0.9	7.5 ± 1.4	9.9 ± 1.4	5.3 ± 1.7	4.1 ± 1.9
T9-C	8.4	10.7 ± 1.2	7.2 ± 1.5	8.1 ± 1.5	3.1 ± 1.3	9.1 ± 1.8	5.3 ± 1.5	4.3 ± 1.3
G10-C	6.6	8.2 ± 1.2	6.0 ± 1.3	6.2 ± 1.5	2.9 ± 1.1	7.2 ± 1.7	6.8 ± 1.0	7.6 ± 1.5
G11-C	7.0	8.0±1.9	7.8 ± 1.4	5.4 ± 1.3	4.9 ± 1.0	7.7 ± 1.3	9.7 ± 0.9	11.1 ± 1.8
T12-C	11.5	10.7 ± 1.5	12.6 ± 1.0	9.6 ± 1.4	10.3 ± 1.3	9.4 ± 1.9	12.8 ± 1.1	14.4 ± 1.4
T13-C	8.7	9.0 ± 1.2	11.7 ± 1.3	12.6 ± 1.6	12.6 ± 1.0	12.6 ± 2.4	16.5 ± 1.4	17.6 ± 1.1
G14-C	7.8	7.7 ± 1.1	10.6 ± 1.5	15.5 ± 2.4	16.6 ± 1.5	15.4 ± 1.9	19.4 ± 1.7	17.9 ± 1.6
G15-C	9.0	7.5 ± 1.2	10.0 ± 2.6	18.4 ± 3.0	19.5 ± 1.6	18.4 ± 2.9	22.8 ± 2.5	22.6 ± 2.8
G1–G15	11.7	8.2 ± 2.5	10.5 ± 2.6	24.0 ± 3.9	26.5 ± 3.6	26.9 ± 4.1	44.3 ± 4.3	43.3 ± 4.3
G2–G14	12.4	10.3 ± 2.1	12.4 ± 2.4	21.5 ± 4.3	26.9 ± 3.5	25.7 ± 3.8	38.3 ± 3.5	35.2 ± 3.1
T3–T13	13.0	15.5 ± 2.5	18.5 ± 2.1	20.9 ± 2.7	23.2 ± 2.3	20.7 ± 4.7	32.1 ± 3.2	33.9 ± 4.2
T4–T12	14.6	16.6 ± 2.6	20.4 ± 2.1	18.8 ± 1.8	18.3 ± 2.0	18.5 ± 3.8	24.8 ± 2.5	27.2 ± 2.7
G5–G11	12.2	13.8 ± 2.3	15.5 ± 2.6	14.4 ± 2.1	14.3 ± 1.4	16.4 ± 2.5	19.1 ± 1.1	21.6 ± 2.0
G6–G10	11.0	11.6 ± 1.2	11.4 ± 1.1	11.0 ± 1.3	10.9 ± 1.1	12.9 ± 1.3	12.6 ± 1.0	13.1 ± 1.6
T7–T9	11.6	11.4 ± 1.3	10.6 ± 0.9	10.9 ± 1.2	11.7 ± 0.9	11.2 ± 1.2	10.5 ± 1.0	12.4 ± 0.8

Standard deviation values (in Å Å) are also displayed.

^a^C represents the centroid of TBA.

^b^Crystal structure of TBA.

^c^The backbone atoms of TBA.

^d^The backbone atoms of TGT loop.

^e^The backbone atoms of T3T4 loop.

^f^The backbone atoms of T12T13 loop.

**Table 3:** Percentage occupation (%) of Hydrogen bonding for seven macrostates in the unfolding pathway from state 3 to state 21 with the maximum flux, derived from 100 representative structures of each state^a^^,^ b.

State-3		State-19	State-5
N7@G14…N2@G2 83%	O6@G5…N2@G11 80%	PO1@T9…N2@G11 80%	PO2@G11…N1@G2 91%
N7@G14…N1@G2 82%	N7@G5…N1@G11 84%	PO1@T9…N1@G11 77%	O3′@G10…N2@G8 85%
N7@G11…N2@G14 76%	O3′@T4…N2@G5 75%	O4′@G6…N2@G2 81%	O6@G5…N1@G6 78%
O6@G11…N2@G14 85%	O6@G2…N2@G5 98%	O5′@G6…N2@G2 83%	N7@G5…N2@G11 81%
O6@G10…N1@G6 93%	N7@G2…N2@G5 90%	O5′@G6…N1@G2 75%	O6@G2…N2@G8 75%
N7@G10…N2@G6 75%	O2@T7…N2@G11 79%	O6@G2…N2@G8 78%	N7@G2…N1@G5 77%
O3′@T9…N2@G10 77%			
**State-24**	**State-11**	**State-6**	**State-21**
N1@G10…N3@T4 83%	O6@G10…N2@G8 85%	O2@T7…N2@G6 51%	O2@T13…N2@G14 54%
O6@G10…N2@G6 87%	PO2@G10…N2@G11 75%	O4@T7…N2@G5 50%	O4@T12…N3@T12 57%
O6@G10…N3@T4 75%	N3@G8…N1@G10 76%		
N7@G10…N1@G6 88%	O2@T7…N2@G6 77%		
N7@G10…N2@G5 86%	N1@G6…N1@G10 79%		
PO2@G10…N2@G11 77%			
O3′@T9…N2@G11 75%			
O3′@T9…N1@G11 78%			
O4@T3…N2@G10 81%			
O4@T3…N1@G10 77%			

^a^For the numbering scheme of the atoms and residues, see Fig. 1.

^b^N7@G14 denotes the N7 atom of the G14 residue and the others are similar.

**Table 4:** Average RMSD values (in Å) with respect to the crystal structure and average distances (in Å) between residues^a^, derived from 100 structures of each state in the unfolding pathway from state 3 to state 6 with the second maximum flux.

State	148D^b^	State-3	State-19	State-16	State-6	State-21
**RMSD/Å**						
All^c^	0	5.0 ± 0.6	8.5 ± 0.6	9.8 ± 0.7	12.6 ± 0.6	12.9 ± 0.6
TGT d	0	3.7 ± 0.7	4.4 ± 0.6	4.1 ± 0.6	4.0 ± 0.6	3.8 ± 0.6
T3T4^e^	0	3.2 ± 0.6	3.3 ± 0.6	4.0 ± 0.6	3.4 ± 0.6	3.4 ± 0.6
T12T13^f^	0	3.7 ± 0.6	3.6 ± 0.6	3.6 ± 0.6	3.7 ± 0.6	3.5 ± 0.6
**Distance/Å**						
G1-C^e^	7.6	7.1 ± 1.3	10.2 ± 4.3	17.5 ± 2.3	22.3 ± 2.0	21.7 ± 2.0
G2-C	8.2	8.3 ± 1.2	11.6 ± 3.0	14.5 ± 2.4	19.4 ± 2.2	18.0 ± 1.8
T3-C	11.0	14.8 ± 1.2	14.5 ± 1.2	13.1 ± 1.9	16.4 ± 1.6	17.4 ± 2.3
T4-C	8.2	9.2 ± 1.4	11.3 ± 0.9	9.7 ± 1.1	12.8 ± 1.6	13.1 ± 1.7
G5-C	7.2	7.5 ± 1.2	9.6 ± 0.8	6.8 ± 1.3	9.8 ± 0.9	10.9 ± 1.4
G6-C	7.3	7.3 ± 1.0	14.1 ± 1.1	8.8 ± 2.2	6.7 ± 1.3	8.4 ± 2.0
T7-C	10.7	10.7 ± 1.6	7.8 ± 0.9	11.1 ± 2.2	9.4 ± 1.8	8.2 ± 1.5
G8-C	8.1	8.0 ± 0.9	6.7 ± 0.8	5.7 ± 1.5	5.3 ± 1.7	4.1 ± 1.9
T9-C	8.4	10.7 ± 1.2	8.4 ± 0.6	6.0 ± 1.9	5.3 ± 1.5	4.3 ± 1.3
G10-C	6.6	8.2 ± 1.2	9.7 ± 0.6	7.9 ± 1.5	6.8 ± 1.0	7.6 ± 1.5
G11-C	7.0	8.0 ± 1.9	11.4 ± 0.7	10.8 ± 1.1	9.7 ± 0.9	11.1 ± 1.8
T12-C	11.5	10.7 ± 1.5	12.7 ± 0.7	13.4 1.0	12.8 ± 1.1	14.4 ± 1.4
T13-C	8.7	9.0 ± 1.2	14.0 ± 1.2	15.6 ± 1.2	16.5 ± 1.4	17.6 ± 1.1
G14-C	7.8	7.7 ± 1.1	10.8 ± 1.3	13.5 ± 2.2	19.4 ± 1.7	17.9 ± 1.6
G15-C	9.0	7.5 ± 1.2	8.8 ± 2.2	12.5 ± 3.6	22.8 ± 2.5	22.6 ± 2.8
G1–G15^g^	11.7	8.2 ± 2.5	11.1 ± 2.1	27.2 ± 4.5	44.3 ± 4.3	43.3 ± 4.3
G2–G14	12.4	10.3 ± 2.1	16.9 ± 5.5	26.1 ± 3.3	38.3 ± 3.5	35.2 ± 3.1
T3–T13	13.0	15.5 ± 2.5	26.1 ± 3.0	27.0 ± 3.4	32.1 ± 3.2	33.9 ± 4.2
T4–T12	14.6	16.6 ± 2.6	23.7 ± 1.54	22.3 ± 2.1	24.8 ± 2.5	27.2 ± 2.7
G5–G11	12.2	13.8 ± 2.3	20.4 ± 1.0	17.2 ± 1.8	19.1 ± 1.1	21.6 ± 2.0
G6–G10	11.0	11.6 ± 1.2	17.9 ± 1.1	13.1 ± 1.9	12.6 ± 1.0	13.1 ± 1.6
T7–T9	11.6	11.4 ± 1.3	9.7 ± 0.6	11.7 ± 1.1	10.5 ± 1.0	12.4 ± 0.8

Standard deviation values are also displayed.

^a^C represents the centroid of TBA.

^b^Crystal structure of TBA.

^c^The backbone atoms of TBA.

^d^The backbone atoms of TGT loop.

^e^The backbone atoms of T3T4 loop.

^f^The backbone atoms of T12T13 loop.

**Table 5:** Percentage occupation (%) of H-bonding for macrostates 7 and 16 in the unfolding pathway from state 3 to state 21 with the second maximum fluxa, b.

State-7		State-16	
N1@G14…N3@T12 78%	N1@G14…N3@T12 78%	O2@T12…N1@G14 77%	O2@T12…N1@G14 77%
O6@G14…N3@T12 75%	O6@G14…N3@T12 75%	O2@T7…N3@T4 78%	O2@T7…N3@T4 78%
O6@G11…N1@G14 83%	O6@G11…N1@G14 83%	N2@G6…N2@G8 81%	N2@G6…N2@G8 81%
N7@G11…N2@G14 87%	N7@G11…N2@G14 87%	PO1@G6…N2@G2 82%	PO1@G6…N2@G2 82%
O6@G10…N2@G14 78%	O6@G10…N2@G14 78%	O3′@G5 …N2@G2 85%	O3′@G5 …N2@G2 85%

^a^For the numbering scheme of the atoms and residues, see Fig. 1.

^b^N1@G14 denotes the N1 atom of G14 residue and the other is similar.
